# Psychometric Properties of the Portuguese Version of the Iceland-Family Perceived Support Questionnaire in Parents of Children and Adolescents with Chronic Condition

**DOI:** 10.3390/ijerph20010247

**Published:** 2022-12-23

**Authors:** Sara Lemos, Luísa Andrade, Maria do Céu Barbieri-Figueiredo, Teresa Martins, Lígia Lima

**Affiliations:** 1 Institute of Biomedical Sciences Abel Salazar, University of Porto, 4050-313 Porto, Portugal; 2 Center for Health Technology and Services Research at the Health Research Network (CINTESIS@RISE), 4200-450 Porto, Portugal; 3 Nursing School of Porto, 4200-072 Porto, Portugal; 4 Nursing Department, University of Huelva, 21071 Huelva, Spain

**Keywords:** family nursing, perceived support, parents, psychometric testing, chronic condition

## Abstract

The support from nurses perceived by family members of children with chronic conditions has been shown to be a protective factor at different levels in a family’s health. As such, nurses need to have instruments that assess this perception to increase the quality of the care provided to those families. This methodological study aimed to analyze the psychometric properties of the Portuguese translation of the Iceland-Family Perceived Support Questionnaire (ICE-FPSQ) in parents of children/adolescents with chronic conditions. The ICE-FPSQ was administered to 237 parents recruited from the day hospital and outpatient services of four hospitals in Northern Portugal. Cronbach’s alpha reliability coefficients for the Total Scale, Cognitive Support, and Emotional Support subscales were excellent (α = 0.96, α = 0.93, α = 0.96, respectively). Reasonable fit indexes were found by confirmatory factor analysis (χ^2^/df = 2.799; CFI = 0.960; PCFI = 0.791, and RMSEA = 0.087), indicating a good model fit to the original structure. The ICE-FPSQ is a valid and reliable instrument to measure perceived support.

## 1. Introduction

Pediatric chronic conditions represent a challenge for the entire family unit, as illness is a family affair [1]. The illness experience impacts families at different levels (physical, psychological, social, and economic) compromising their quality of life [2,3]. Family functioning is affected as well as the quality of parenting practices when a child is sick [4].

The frequent use of health services by families of children/adolescents with chronic conditions, whether for consultations, treatments, or hospitalizations, offers nurses the opportunity to identify family needs and intervene accordingly [5,6]. It is important that the family feels supported and acknowledged. In this context, the support nurses offer plays a facilitating role in a family’s adaptation to the pediatric chronic condition [2,7]. Given the diversity of families, this support should be adjusted to their perceived difficulties, their needs, and specific health–illness situation [1]. 

Health professionals often believe that their interventions meet a family’s needs [8]. Nevertheless, health professionals’ judgment benefits from an accurate assessment of family members’ perception of the support they receive [5]. To this end, Sveinbjarnardottir, Svavarsdottir, and Hrafnkelsson [9] developed the Icelandic-Family Perceived Support Questionnaire (ICE-FPSQ) to assess family members’ perceptions of nurses’ support. 

This instrument is conceptually founded on the Calgary Family Intervention Model (CFIM) [10]. According to this approach, interventions directed to the affective domain focus on validating/normalizing the family’s emotional responses, promoting family support, closely monitoring the concerns and feelings of each family member, and identifying resources and strengths for mutual support. They also highlight the importance of encouraging narratives about the illness, sharing stories about the illness and associated suffering, and the stories of strength and resilience. Interventions associated with the cognitive domain of family functioning encompass information sharing, decision-making support, and empowering the family to adopt coping strategies to deal with the illness, praising the competence and strengths of the family and its members by routinely including commendations on family behavior [1,10].

As an instrument that assesses family members’ perceptions, it has been used with different populations in several settings and countries, such as families of patients undergoing cancer treatment in Australia [11], family members and adults with chronic disease in Denmark [12], families living with mental illness in Norway [13], family members and adults diagnosed with depression in Portugal [14], family members of patients admitted to the emergency department in South Africa [5], parents of children with cardiac congenital heart defects in Sweden [15], family members of critically ill adults in Switzerland [16], and with adult patients from acute care settings in United States [17].

According to studies on the development of nursing interventions focused on family support, interventions oriented toward the family’s cognitive and emotional domains have shown benefits for the family unit [6,18]. In addition, perceived support of family members of children with chronic illness from healthcare professionals has shown to be a protective factor at different levels, namely reducing family stress, and better parenting practices, well-being, and adaptation to the chronic condition [2,4,19].

Given the usefulness and importance of having an instrument to assess a family’s perception of nurses’ support in parents of children/adolescents with chronic illness, this study aims to analyze the psychometric properties of the Portuguese version of the ICE-FPSQ [14] in this specific population.

## 2. Materials and Methods

A methodological study was conducted from May 2021 to January 2022.

### 2.1. Sample

This study involved a convenience sample of 237 voluntary participants who were parents of pediatric patients with chronic conditions. These chronic conditions encompassed diseases such as those in the metabolic/endocrine system (e.g., diabetes) or respiratory system (e.g., asthma), as well as psychological disorders (e.g., generalized anxiety disorder), and developmental disorders (e.g., cerebral palsy). The decision to include all these conditions was based on the assumption that they all have lifelong effects on the functionality of the child/adolescent and their families. Participants were recruited from the outpatient clinics and pediatric day hospitals of four Hospital Institutions in Northern Portugal. The inclusion criteria were: parents of children/adolescents with one or more diagnoses of chronic disease and/or chronic disorders; diagnosed more than six months prior to the study; proficient in the Portuguese language; and access to an electronic device with the internet. 

### 2.2. Data Collection

In total, 507 parents were recruited and 237 parents completed all instrument items and were included in the analysis (44.9%). To study the temporal stability of the instrument, all parents were invited to answer the questionnaire twice; however, only 46 agreed to participate in the second measurement. Of these 46, 10 did not correctly fill out the identification code so it was not possible to match the two measurements. The invitation to complete the second measurement was sent one week after the first, and questionnaires were filled out between 7 and 26 days later.

Data were collected through an online questionnaire using Research Electronic Data Capture (REDCap) (Vanderbilt University, Nashville, TN, USA) [20]. Demographic data included the parents’ ages, genders, educational level, marital status, and employment status, as well as children/adolescent-related variables including gender, age, and diagnosis.

### 2.3. Iceland-Family Perceived Support Questionnaire (ICE-FPSQ)

The ICE-FPSQ was developed by Sveinbjarnardottir et al. [9] in Iceland. The scale is a self-reported questionnaire with 14 items, which assesses families’ perceived support from nurses. Specifically, it considers (a) cognitive support (five items), and (b) emotional support (nine items). It uses a 5-point Likert-type scale ranging from 1 (almost never) to 5 (almost always). The total score from ICE-FPSQ ranges from 5 to 70. The cognitive support subscale ranges from 5 to 25, and the emotional support subscale from 9 to 45 points. Higher scores reflect a perception of greater support offered by the nurse. The reliability of the ICE-FPSQ total score and each of its two factors were very good to excellent (α = 0.96 for total scale; cognitive support subscale, α = 0.88; and for emotional support subscale, α = 0.95) [9]. In Portugal, the scale was translated and adapted for the Portuguese population using a sample of 119 members of Portuguese families of adults with depression, and showed excellent internal consistency (α = 0.94) [14]. In this study, the analysis suggested the exclusion of one item (item 8). Nevertheless, as we considered this item particularly important and useful in assessing support provided by nurses to families of children and adolescents we decided to include this item in the version studied here.

### 2.4. Ethical Considerations 

This study was approved by all four Ethics Committees of the Hospital Institutions of Northern Portugal involved in this study (No. 52/2021, No. 38/2021, No. 98_2021, and No. 16/2021). All participants were informed about the study objectives and provided with an informed consent form. After they accepted to participate in this study they were sent an email with a link to REDCap Software)(Vanderbilt University, Nashville, TN, USA) to complete the questionnaire [20].

### 2.5. Analysis 

The program IBM-SPSS version 28.0 was used for data analysis. The analysis was performed using descriptive measures, namely mean, standard deviation, asymmetry, and kurtosis. In the exploratory analysis of the ICE-FPSQ, the distribution of each item and the missing values were analyzed.

For the internal consistency analysis, the Cronbach’s α coefficient was calculated to evaluate the homogeneity of the scale. Good internal consistency is reached with an alpha value higher than 0.80, but lower values are acceptable when a very low number of items are analyzed [21]. The test–retest analysis was conducted by intraclass correlation (ICC). The ICC coefficient is expected to be above 0.7 for satisfactory stability over time [22].

The confirmatory factor analysis was conducted through IBM-AMOS (version 27) software to evaluate the bifactorial structure of ICE-FPSQ. 

The existence of outliers was evaluated by the square of the Mahalanobis distance, and normality was calculated through the coefficient of asymmetry and uni- and multivariate kurtosis. The covariance matrix was inputted, and the method of Maximum Likelihood estimates was used. The quality of the global fit of the factorial model was assessed according to the indices and respective reference values [23,24]. We additionally assessed factor loadings, communalities, and factor reliability as local fit indices. The chi-square test (χ^2^/df), the comparative fit index (CFI), the parsimony comparative fit index (PCFI), and the root mean square error of approximation (RMSEA, *p* [rmsea ≤ 0.05]) were used. For model fit, the chi-square value (CMIN/DF) is recommended to be lower than 3. The CFI need to be close to 0.90. The PCFI must show values above 0.60, while the recommended RMSEA is up to 0.08 [23,24]. 

## 3. Results

### 3.1. Descriptive Results 

The majority of the 237 parents that participated in this study were aged between 41 and 50 years of age (56.1%) and the majority were female (87.3%). Most (81.8%) had a partner (i.e., married or cohabiting) and had completed secondary education (37.6%) or higher education (32.9%). Approximately half of the parents (57.4%) worked full time.

Regarding the children, most were in the 11-18 years old age group (53.5%), and 121 (51.1%) were male. The most common conditions affecting children were the group of chronic conditions (81.3%) (i.e., respiratory, or gastrointestinal problems), followed by psychological disorders (16.7%), and developmental disorders (2.0%). Some children/adolescents had been diagnosed with more than one disease and/or disorder (Table 1).

Results obtained from the ICE-FPSQ are shown in Table 2. The answers showed considerable variation in the parents’ perception of support provided by nurses. Nevertheless, in item 6 (… offered us family meetings) and item 12 (… encouraged my family to take a respite from caregiving) it is possible to observe a floor effect as more than 60% of the responses are situated in the option “almost never”.

Table 3 shows the mean scores for each item of the subscales and the total score, as well the analysis of asymmetry and kurtosis. 

### 3.2. Reliability and Temporal Stability

The internal consistency for the Total Scale, and the Cognitive Support and Emotional Support subscales were calculated. The alpha values found were excellent (α = 0.96, α = 0.93, α = 0.96, respectively). For each subscale, the internal consistency analysis also showed that the alpha value would not increase if any items were deleted, so it was decided to maintain them all. 

The parents that participated in the test–retest analysis had sociodemographic characteristics similar to the total sample. In addition, ICC_S_ analysis for both subscales and the Total Scale showed satisfactory stability over time (Table 4). 

### 3.3. Construct Validity—Confirmatory Factor Analysis

The factorial model tested was adjusted to the sample, although it revealed some poor indices (χ^2^ = 264.415; *p* < 0.001; χ^2^/df = 3.479; CFI = 0.944; PCFI = 0.789 and RMSEA = 0.102). To improve the fit of the model, seven observations were excluded as values of D2 (exploration of Mahalanobis distances) suggesting the presence of outliers (*p*1 and *p*2 < 0.001). Based on the modification indices, trajectories were also included in the model between the pairs of item residues item 13 and item 14. As such, a reasonable fit was reached (χ^2^ = 209.973; *p* < 0.001; χ^2^/df = 2.799; CFI = 0.960; PCFI = 0.791, and RMSEA = 0.087).

**Figure 1:**
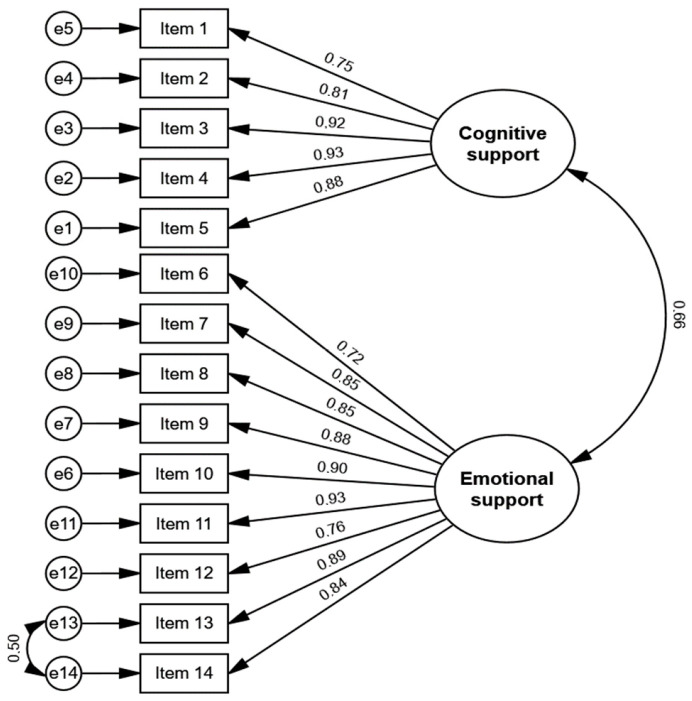
Confirmatory Factory Analysis of the Portuguese version of Iceland-Family Perceived Support Questionnaire.

Figure 1 shows that the Portuguese version confirms the factorial structure proposed by the original authors of the ICE-FPSQ scale. 

## 4. Discussion

The results of this study confirm the validity and reliability of the European Portuguese version of the ICE-FPSQ in parents of children/adolescents with chronic conditions. 

The analysis of possible response options showed that in the majority of the items all five response categories were endorsed, and there was also a small percentage of missing values. A floor effect was found in items 6 (“The nurses on the unit have offered us family meetings”) and 12 (“The nurses on the unit have encouraged my family to take a respite from caregiving”) in the response option “almost never”. This finding suggests that current practice in nursing interventions is not reflected for these two items in this setting, ambulatory pediatric services, as it happens in other countries where the practice of advanced family nursing is already established [18]. These two items belong to the emotional support subscale [9], suggesting that this is probably an area less developed in nursing care and less valued by parents. In the study conducted in Sweden with families of children with heart defects, those two items also had the lowest scores [15]. 

We should take into consideration that the ICE-FPSQ was developed to assess families’ perception of the support provided by nurses based on advanced family nursing practice [9]. This advanced practice does not yet happen in the Portuguese context but is desirable to be implemented, and continuing education is required. A recent systematic review identified education “as an effective tool in increasing the confidence of nursing in working with families and patients in the areas of knowledge, skill, comfort, family systems, assessment, and interactions” [7] (p. 1342).

The internal consistency reliability of the scale was evaluated by Cronbach’s alpha coefficients. All Cronbach’s alpha coefficients exceeded the recommended standard of 0.80 for the Total Score, the Cognitive Support Subscale, and the Emotional Support Subscale, indicating excellent reliability of the ICE-FPSQ Portuguese version. The results obtained in this study corroborate those presented in the original version by Sveinbjarnardottir and colleagues [9], and in the study by Bruce and colleagues with parents of children with cardiac congenital heart defects in Sweden [15]. These results were similar to the internal consistency values obtained in other versions, such as the study with families of patients undergoing cancer treatment in Australia [11], family members and adults with chronic disease in Denmark [12], families living with mental illness in Norway [13], family members and adults diagnosed with depression in Portugal [14], and family members of patients admitted to the emergency department in South Africa [5].

Test–retest reliability was examined using ICC coefficient and the value for the Total Scale was 0.89. Thus, our results showed a good test–retest reliability of the scale, as it was higher than 0.70 [21] indicating it is a stable measuring instrument when administered under the same conditions and with the same participants at different times. These results are supported by the studies conducted by Swedish researchers with parents of children with cardiac congenital heart defects in 2016 [15] and Danish researchers with family members and adults with chronic disease in 2018 [12].

The evaluation of the validity of the Portuguese version of the ICE-FPSQ showed that the original version of the model [8] had a poor fit. To improve the model fit, seven observations were excluded as the presence of outliers (*p*1 and *p*2 < 0.001) was suggested, and a correlation between the pairs of residues of items 13 and 14 in the model was included. As such, a reasonable fit was reached for the Portuguese version and confirmed the factorial structure proposed by the original authors of the ICE-FPSQ scale. The structural analysis was almost the same as the original version [9], the Swedish translation [15], and the Danish translation [12]. 

The RMSEA was found to be 0.09, indicating acceptable values as the recommended value is up to 0.08 [23,24]. In the original scale, the value was 0.04 [9], and the Danish [12] and Swedish versions were 0.12 [15]. These differences might be explained by differences in cultural settings and populations [12]. Moreover, the RSMEA value tends to be higher in structurally simple models with few degrees of freedom [23,25].

The study is considered to have the following limitations. First, we did not include instruments to evaluate the convergent and divergent validity. Another limitation was the difficulty of getting more responses from participants for the test–retest evaluation. 

## 5. Conclusions

There is a move toward the provision of family-centered care in pediatric settings, and to be consolidated, instruments are needed to assess nurses’ practice. 

This study aimed to evaluate the psychometric properties of the Portuguese version of the ICE-FPSQ translated to European Portuguese in parents of children/adolescents with chronic conditions. The Portuguese version of the ICE-FPSQ was found to be a valid and reliable instrument to measure the families’ perceptions of the support provided by nurses in the context of pediatric chronic conditions. 

The use of this instrument will be useful in a range of healthcare institutions to improve the quality of family-centered care, as it is an important and promising tool for nurses to assess the effectiveness of evidence-based family interventions.

## Figures and Tables

**Table 1:** Descriptive characteristics of the sample (*n* = 237).

Demographic Characteristics	*n*	%
Participants Gender		
Male	28	11.8
Female	207	87.3
No answer	2	0.8
Age (years)		
18 to 30	8	3.4
31 to 40	73	30.8
41 to 50	133	56.1
51 to 60	20	8.4
≥61	3	1.3
Education level		
Elementary School	37	15.6
Secondary Education	89	37.6
Professional course	14	5.9
University degree	78	32.9
Other	19	8.0
Marital status		
Married/cohabiting	194	81.8
Single/divorced	36	15.2
Widowed	6	2.5
No answer	1	0.4
Employment status		
Full-time	136	57.4
Part-time	19	8.0
Unemployed	27	11.4
Full-time and second job	12	5.2
Other	43	18.0
Children/Adolescent		
Gender		
Female	114	48.1
Male	121	51.1
Age (years)		
1–3	32	14.0
4–6	23	10.1
7–10	51	22.4
11–18	122	53.5
Chronic Condition		
Respiratory system	49	16.3
Gastrointestinal tract	37	12.3
Metabolic/endocrine system	32	10.7
Neurological system	32	10.7
Syndromes	30	10.0
Immunological system	22	7.3
Urological system	16	5.3
Cardiovascular system	11	3.7
Dermatology	5	1.7
Oncology	4	1.3
Ophthalmology	3	1.0
Hematological system	2	0.7
Otolaryngology	1	0.3
Psychological disorder	50	16.7
Developmental disorder	6	2.0

**Table 2:** Response Frequencies to the Items of the Portuguese version of ICE-FPSQ (*n* = 237).

Item	The Nurses on the Unit Have…	Results *n* (%)					Missing *n* (%)
		Almost Never	Rarely	Sometimes	Usually	Almost Always	
1	… offered us information and their professional opinion	16 (6.8)	18 (7.6)	31 (13.1)	84 (35.4)	87 (36.7)	1 (0.4)
2	… provided accessible and easy-to-read literature about the health problem	43 (18.1)	27 (11.4)	33 (13.9)	64 (27.0)	66 (27.8)	4 (1.7)
3	… informed my family about the resources available in the community that have proven to be helpful for families in similar situations	55 (23.2)	36 (15.2)	39 (16.5)	51 (21.5)	54 (22.8)	2 (0.8)
4	… provided ideas, information, and thoughts in a manner that enabled us to learn from them and reflect on them	49 (20.7)	33 (13.9)	42 (17.7)	60 (25.3)	50 (21.1)	3 (1.3)
5	… emphasized the use of family rituals to promote family members’ health	40 (17.7)	28 (11.8)	52 (21.9)	61 (25.7)	54 (22.8)	2 (0.8)
6	… offered us family meetings	153 (64.6)	31 (13.1)	23 (9.7)	15 (6.3)	11 (4.6)	4 (1.7)
7	… helped family members recognize that our emotional response is valid and helped us to validate and/or normalize family members’ emotional response	99 (41.8)	49 (20.7)	34 (14.3)	28 (11.8)	25 (10.5)	2 (0.8)
8	… encouraged my family to become involved with the healthcare team in the care of our family member and have offered us caregiver support	77 (32.5)	38 (16.0)	40 (17.7)	44 (18.6)	36 (15.2)	2 (0.8)
9	… encouraged family members to share their illness narratives—not only stories of illnesses and suffering but also stories of strength and resilience	110 (46.4)	33 (13.9)	38 (16.0)	29 (12.2)	24 (10.1)	3 (1.3)
10	… drawn out our family strengths	87 (36.7)	37 (15.6)	32 (13.5)	40 (16.9)	38 (16.0)	3 (1.3)
11	… helped family members understand how our emotional response is related to the family member’s illness	95 (40.1)	42 (17.7)	28 (11.8)	39 (16.5)	29 (12.2)	4 (1.7)
12	… encouraged my family to take a respite from caregiving	141 (59.5)	33 (13.9)	15 (6.3)	25 (10.5)	19 (8.0)	4 (1.7)
13	… been aware of the impact family members can have on one another, on the patient’s well-being, and on the illness itself	97 (40.9)	40 (17.7)	34 (14.3)	36 (15.2)	27 (11.4)	3 (1.3)
14	… looked for the family’s strengths and opportunities to commend family members when their strengths have been revealed	102 (43.0)	39 (16.5)	28 (11.8)	38 (16.0)	27 (11.4)	3 (1.3)

**Table 3:** Descriptive statistics of the items of the Portuguese version of ICE-FPSQ (*n* = 237).

Item	*The Nurses on the Unit Have…*	*M (SD)*	*Sk*	*Ku*
1	… offered us information and their professional opinion	3.88 (1.18)	−1.037	0.247
2	… provided accessible and easy-to-read literature about the health problem	3.35 (1.45)	−0.432	−1.185
3	… informed my family about the resources available in the community that have proven to be helpful for families in similar situations	3.06 (1.49)	−0.103	−1.407
4	… provided ideas, information and thoughts in a manner that enabled us to learn from them and reflect on them	3.12 (1.43)	−0.208	−1.289
5	… emphasized the use of family rituals to promote family members’ health	3.26 (1.38)	−0.335	−1.089
6	… offered us family meetings	1.72 (1.16)	1.545	1.294
7	… helped family members recognize that our emotional response is valid and helped us to validate and/or normalize family members’ emotional response	2.28 (1.38)	0.729	−0.794
8	… encouraged my family to become involved with the healthcare team in the care of our family member and have offered us caregiver support	2.68 (1.47)	0.237	−1.362
9	… encouraged family members to share their illness narratives—not only stories of illnesses and suffering but also stories of strength and resilience	2.25 (1.40)	0.713	−0.880
10	… drawn out our family strengths	2.60 (1.51)	0.340	−1.379
11	… helped family members understand how our emotional response is related to the family member’s illness	2.41 (1.45)	0.544	−1.167
12	… encouraged my family to take a respite from caregiving	1.92 (1.34)	1.217	0.037
13	… been aware of the impact family members can have on one another, on the patient’s well-being, and on the illness itself	2.38 (1.43)	0.568	−1.096
14	… looked for the family’s strengths and opportunities to commend family members when their strengths have been revealed	2.35 (1.45)	0.600	−1.112
**Cognitive Support** **subscale**		3.33 (1.24)	0.398	−0.970
**Emotional Support** **subscale**		2.29 (1.22)	−0.668	−0.716
**Total Scale**		2.66 (1.11)	0.630	−0.699

Note. *M*: mean; *SD*: standard deviation; *Ku*: Kurtosis; *Sk*: skewness.

**Table 4:** Test–retest analysis of the ICE-FPSQ (*n* = 36).

Intraclass Correlation Coefficient ^a^ (95% CI)			
ICE-FPSQ		95% CI	
	ICC ^b,c^	Lower Bound	Upper Bound
**Cognitive Support**	0.90	0.79	0.95
**Emotional Support**	0.81	0.66	0.90
**Total Scale**	0.89	0.78	0.95

Note. CI: confidence interval; ICC: intraclass correlation. Two-way random effects model where people are random and measures effects are fixed. ^a^ Type A intraclass correlation coefficient using an absolute agreement definition. ^b^ This estimate is computed assuming the interaction effect is absent because it is not estimable otherwise. ^c^ Average measures.

## Data Availability

The data that support the findings of this study are available from the corresponding author upon reasonable request.
